# Shunt fracture in two children with myelomeningocele following spine surgery

**DOI:** 10.4103/2152-7806.70852

**Published:** 2010-10-06

**Authors:** Nazanin Baradaran, Farideh Nejat, Nima Baradaran, Mostafa El Khashab

**Affiliations:** Department of Neurosurgery, Children’s Hospital Medical Center, Tehran University of Medical Sciences, Iran; 1 Department of Neurosurgery, Hackensack University Medical Center, New Jersey, and Section of Pediatric Neurosurgery, Saint Barnabas Medical Center, Livingston, New Jersey, USA

Sir,

Most neonates with myelomeningocele (MMC) develop symptomatic hydrocephalus within the first 6 weeks of life. Ventriculoperitoneal (VP) shunt surgery is the most frequent operation, which is used in order to treat hydrocephalus.[11]

Shunt failure rate is estimated as high as 40% by 1 year and 50% by 2 years and 70% after the fifth year.[2,5,6] According to current data, approximately 56–80% of the patients experience at least one episode of malfunction during the 10 years following insertion.[8,10] The non-infectious causes of shunt failure include obstruction, overdrainage, loculation of the ventricles, abdominal complications and mechanical failure including fracture and kinking of the tube, disconnection of components, migration of the shunt or misplacement.[9]

The typical presentation of a fractured shunt system is usually quite late after initial insertion and it may be marked by the rapid onset of dramatic symptoms such as headache, nausea, swelling over the shunt tract often in a location over the shunt fracture or it may occur in a more subtle fashion over a longer period of time.[1] Here, we report two children with shunted hydrocephalus and history of MMC who presented with acute shunt malfunction related to distal tube fracture shortly following the spinal surgery.

## CASE DESCRIPTION

### Case 1

**Figure 1:**
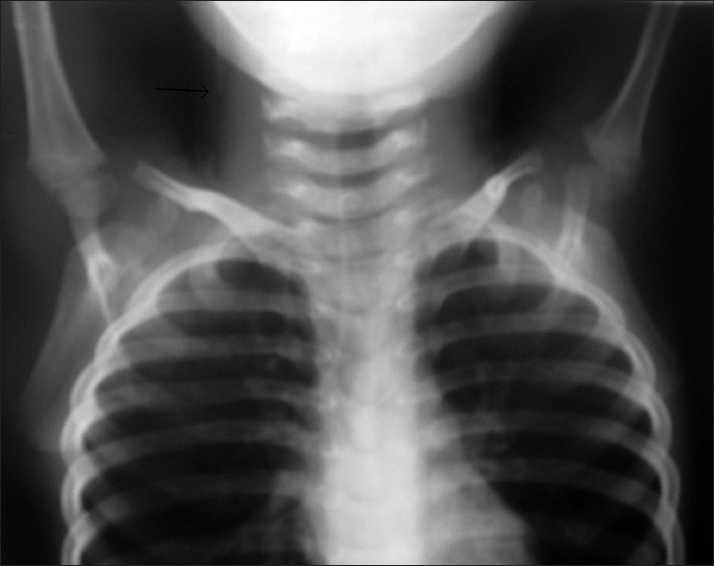
Chest X-ray of the patient shows no catheter in this view and cut of the catheter at the neck

The patient is a 6-year-old girl who was initially referred to our neurosurgical service at the age of 2 months with hydrocephalus and MMC. She underwent successful VP shunt insertion and MMC repair. She was regularly monitored for her shunt function, developmental milestones as well as urological and orthopedic problems. Six years thereafter, she developed new-onset leg and back pain and progressive worsening of her gait, which her magnetic resonance imaging (MRI) confirmed post-surgical tethered cord with spinal cord adherence to previous MMC surgery scar. Plain X-ray shunt series performed during the admission time showed no breakage of shunt catheter. She underwent an untethering procedure in the prone position with her head turned to one side on the doughnut without any operative complications; however, she was readmitted 10 days later because of a 2-day history of severe headache and vomiting leading to drowsiness without any back wound problems. On examination, resistance against shunt flushing was observed with simultaneous subcutaneous swelling on her neck. New shunt series showed the absence of radioopaque catheter tract in her chest [Figure 1] while both ends of the tube were found in the abdomen. She was urgently managed with standard procedure for new distal catheter insertion. The distal peritoneal shunt was broken at a 12-cm distance from the connection point to the pump. Her post-operative course was unremarkable.

### Case 2

**Figure 2:**
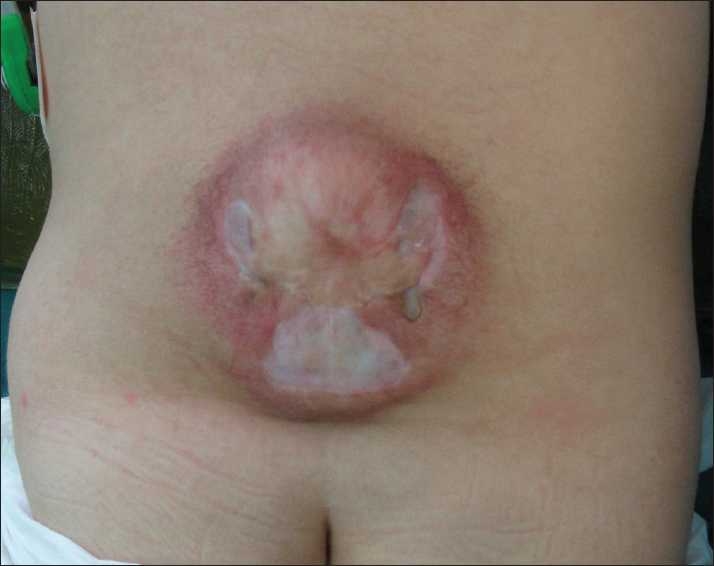
Photography from the back shows the scarred myelomeningocele sac

A 5-year-old boy was admitted due to a 7-day history of cerebrospinal fluid (CSF) leakage from an unoperated MMC wound. He had undergone VP shunt insertion at the age of 6 months due to hydrocephalus leading to gross head enlargement and symptomatic intracranial hypertension. At that time, his parents had refused MMC repair due to severe neurological deficit. His follow-up was not regular. Two years thereafter, he was referred to us for proximal shunt malfunction that was surgically managed. Upon his recent admission, he was managed while putting him on prone position for CSF leakage from his MMC wound at the place of a thin scar on the tip of the thoracolumbar kyphosis [Figure 2]. On examination, a well-functioning shunt with good filling and emptying of the pump was detected and confirmed by the brain computed tomography (CT) scan. Plain X-ray at this time showed intact catheter in its tract from the skull to the peritoneum. Primary repair of MMC and watertight dural repair were performed in the standard manner in the presence of severe scar and tissue adhesions. After 4 days, he demonstrated headache and CSF collection at the place of MMC surgery. Flushing of the pump was associated with pain and swelling in his neck. A new brain CT scan revealed enlargement in ventricular size while plain X-ray showed presence of the whole distal tube in the abdomen. He underwent surgery with distal catheter revision. The distal peritoneal shunt tubing was broken in the neck about 8 cm away from the pump. The patient did well post-operatively and his CSF collection disappeared in the presence of good functioning revised shunt.

## DISCUSSION

Shunted hydrocephalus is a condition that prompts continuous medical surveillance and surgical intervention due to a vigilant fear of malfunction and infection. At least one shunt revision surgery is required in about half of the patients shunted due to MMC during the first year.[7] More shunt complications occur in children with higher spinal lesions and more severe hydrocephalus.[11] Shunt dysfunction could result from a broad spectrum of factors such as suboptimal surgical techniques, isolated shunt material failure or patient-related factors like concurrent medical illnesses. However, in a majority of the cases, multiple factors are involved.[4]

The risk of shunt fracture is higher in children[11] and this might be due to the ongoing pressure on a part of a shunt located between two fixation points in the growing child. The most common location for a fracture is along the distal catheter segment, often near the clavicle or over the lower ribs.[1] Mechanical stresses, such as lengthening during deformity correction along with calcification and tethering of the tube, predispose the shunt catheter to fracture.[3]

In our patients with well-functioning pre-operative shunts, prone position during and after the surgery in order to decrease the risk of wound CSF leakage could be assumed as the main cause for this failure. The positioning of the patient under general anaesthesia can provide a kind of mechanical stress on the catheter that when the child is awake, it is impossible to be reproduced due to pain and probable contracture. Moreover, the shunt might be prone to fracture by previous chest deformity associated with the preceding calcification of the catheter, as seen in case 2. Attentive follow-up of the shunt function after any spine surgery in the shunted patients is advised.

## CONCLUSION

VP shunting for hydrocephalus has come to stay as the predominant treatment, and malfunction is one of the most common clinical problems encountered in pediatric neurosurgery. Here, we describe two patients with shunted hydrocephalus who presented with acute shunt malfunction related to distal tube fracture just following a spinal surgery. Prone position during surgery and afterwards and associated chest deformity could be anticipated as the main causes of this complication.
